# Unravelling the Interactions between Hydrolytic and Oxidative Enzymes in Degradation of Lignocellulosic Biomass by* Sporothrix carnis* under Various Fermentation Conditions

**DOI:** 10.1155/2016/1614370

**Published:** 2016-01-11

**Authors:** Olusola A. Ogunyewo, Folasade M. Olajuyigbe

**Affiliations:** Department of Biochemistry, Federal University of Technology, Akure 340001, Nigeria

## Abstract

The mechanism underlying the action of lignocellulolytic enzymes in biodegradation of lignocellulosic biomass remains unclear; hence, it is crucial to investigate enzymatic interactions involved in the process. In this study, degradation of corn cob by* Sporothrix carnis* and involvement of lignocellulolytic enzymes in biodegradation were investigated over 240 h cultivation period. About 60% degradation of corn cob was achieved by* S. carnis* at the end of fermentation. The yields of hydrolytic enzymes, cellulase and xylanase, were higher than oxidative enzymes, laccase and peroxidase, over 144 h fermentation period. Maximum yields of cellulase (854.4 U/mg) and xylanase (789.6 U/mg) were at 96 and 144 h, respectively. Laccase and peroxidase were produced cooperatively with maximum yields of 489.06 U/mg and 585.39 U/mg at 144 h. Drastic decline in production of cellulase at 144 h (242.01 U/mg) and xylanase at 192 h (192.2 U/mg) indicates that they play initial roles in biodegradation of lignocellulosic biomass while laccase and peroxidase play later roles. Optimal degradation of corn cob (76.6%) and production of hydrolytic and oxidative enzymes were achieved with 2.5% inoculum at pH 6.0. Results suggest synergy in interactions between the hydrolytic and oxidative enzymes which can be optimized for improved biodegradation.

## 1. Introduction

Lignocellulosic biomass which is considered to be the mass of organic material from any biological origin or matter has a wide variety of resources available for conversion into bioproducts [1]. These resources can be utilized to create new biomaterials such as fuels, chemicals, animal feeds, soil conditioners, and fertilizers [2]. However, underutilization or ineffective management of these biomasses results in environmental pollution on the earth surface putting humans at various risks [3].

Chemical method of hydrolysis had previously been used for recovery of cellulose from lignocellulosic biomasses but the method is highly expensive and energy intensive as it involves mechanical treatment with acid, alkali, and steam explosion [4, 5]. This major challenge therefore makes the search for better and efficient method for hydrolyzing lignocellulose through biological means very crucial with the production of lignocellulolytic enzymes.

Cellulase, xylanase, laccase, and peroxidases are extracellular lignocellulolytic enzymes capable of catalysing the biodegradation of lignocellulose. Cellulase, one of the hydrolytic enzymes involved in degradation of lignocellulose, consists of endoglucanases, exoglucanases, and *β*-glucosidases which catalyses the hydrolysis of cellulose, a linear polysaccharide polymer with many glucose monosaccharide units not only for liquid fuel production but also for the production of other chemicals which can be potential substitutes for petroleum derivatives [6, 7]. Xylanase (endo-1,4-*β*-D-xylanohydrolase) plays important role in depolymerisation of xylan, the main renewable hemicellulosic polysaccharide of plant cell wall. It catalyses the degradation of xylan by cleaving the xylosyl backbone and releasing short xylooligosaccharides, which are further hydrolyzed into xylose units by xylan 1,4-*β*-xylosidase [8, 9]. Laccases and peroxidases are known oxidoreductive enzymes which are considered to be most effective in the removal of the lignin component of lignocellulose. These lignocellulolytic enzymes have been reported to possess wide applications in textile, paper and pulp, food, chemical, and biobleaching industries and also in biofuel industry for energy generation [3, 10, 11].

Studies have shown that certain species of white rot fungi (WRF) could achieve efficient biodegradation of lignocellulose with the production of industrially useful oxidoreductive and hydrolytic enzymes when cultivated in appropriate media containing a source of carbon and nitrogen that can stimulate their growth and multiplication [12–14]. WRF are not only capable of producing lignocellulolytic enzymes but also able to penetrate the substrate to transport these enzymes into materials such as wood chips by hyphal extension [15]. However, there is dearth of information on the interaction of lignocellulolytic enzymes from fungal strains in the degradation of lignocellulosic biomass. Furthermore, the mechanism underlying the interaction of the lignocellulolytic enzymes for improved biodegradation of lignocellulose by white rot fungi is unclear. Hence, there is need to examine the interactions of the hydrolytic and oxidative enzymes involved in lignocellulose degradation and the impact of the interaction on the degradation process [14, 16] under different fermentation conditions. In this study, we investigated the effect of some process parameters on production of lignocellulolytic enzymes by a white rot fungus and examined how these enzymes interact with one another for improved biodegradation of lignocellulose.

## 2. Materials and Methods

### 2.1. Materials

Carboxymethyl cellulose, birch wood xylan, 2,2′-azino-di-[3-ethylbenzothiazoline-6-sulphonic acid] (ABTS), dinitrosalicylic acid, sodium-potassium tartrate, bovine serum albumin (BSA), manganese sulphate, copper sulphate, tyrosine, leucine, cellobiose, avicel, xylose, tryptophan, aspartate, glutamate, hydrogen peroxide, and media components were products of Sigma-Aldrich (St Louis, MO, USA). Corn cob was purchased from a local market. The corn cob was sun-dried and powdered into fine particles which was utilized as carbon source in the basal media. Further processing on the powdered corn cob was carried out using standard sieve to an average size of 1 mm. All other chemicals used were of analytical grade.

### 2.2. Fungal Strain

The microorganism used was a white rot fungus isolated from decaying wood in a selected citrus plantation in Ijare, Ondo State, South West Nigeria. This strain was identified as* Sporothrix carnis* by the Biotechnology Unit of Federal Institute of Industrial Research, Lagos, based on morphological and biochemical methods described by Collins et al. [17]. The fungal strain was maintained on fresh potato dextrose agar (PDA) slants and stored at 4°C.

### 2.3. Inoculum Preparation and Production of Lignocellulolytic Enzymes

Seed culture was prepared by growing a loopful of slant culture in 30 mL culture medium containing glucose (10.0 g/L), ammonium nitrate (2.0 g/L), KH_2_PO_4_ (0.8 g/L), K_2_HPO_4_ (0.2 g/L), MgSO_4_·7H_2_O (0.5 g/L), and yeast extract (2.0 g/L) in a 200 mL conical flask with pH adjusted to 6.0 [10]. The culture was incubated at 30°C for 72 hr at 160 rpm in a shaking incubator (Stuart, UK). The 3-day-old seed culture was used as inoculum for the production media. Seed inoculum of 1.5 mL (constituting 3% v/v) was transferred into a 50 mL corn cob based (CCB) media which comprises corn cob (10 g/L), ammonium nitrate (2.0 g/L), KH_2_PO_4_ (0.8 g/L), K_2_HPO_4_ (0.2 g/L), MgSO_4_·7H_2_O (0.5 g/L), CuSO_4_·5H_2_O (0.25 g/L), yeast extract (2.0 g/L), and MnSO_4_·7H_2_O at pH 6.0. At the end of 192-hour incubation period, cultures were harvested by centrifugation at 10,000 rpm for 15 min at 4°C using refrigerated benchtop centrifuge (Eppendorf 5810R). The cell free supernatant was recovered as crude enzyme preparation and assayed for the presence of cellulase, xylanase, laccase, and total peroxidase.

### 2.4. Enzyme Assays

#### 2.4.1. Determination of Cellulase Activity

The supernatants obtained after biomass separation were analyzed for enzyme activity. Endoglucanase (CMCase) activity was determined using the method described by Wood and Bhat [18] with some modifications. One hundred and fifty microliters (150 *μ*L) of enzyme extract was added to 450 *μ*L of 1% (w/v) carboxymethyl cellulose (CMC) in 50 mM sodium acetate buffer (pH 4.8) in an Eppendorf tube and incubated at 40°C for 20 minutes. The reaction was terminated with the addition of 400 *μ*L of dinitrosalicylic acid (DNSA) and boiled at 100°C for 5 min. The absorbance was recorded at 575 nm against blank. One unit of CMCase activity was expressed as 1 *μ*mole of glucose liberated per minute under standard assay conditions.

#### 2.4.2. Determination of Xylanase Activity

Xylanase activity was determined according to the method of Saha [19] with slight modification. The reaction mixture comprised 400 *μ*L of 1% (w/v) solution of birch wood xylan in 50 mM Tris-HCl buffer pH 9.0 incubated with 100 *μ*L of culture supernatant for 15 min at 40°C. The released reducing sugar was assayed using the DNSA method [20]. One unit of xylanase activity was defined as the amount of the enzyme that liberated 1 *μ*mol of xylose equivalent per minute under the standard assay conditions.

#### 2.4.3. Determination of Laccase Activity

Laccase activity was determined according to a modified method of Bourbonnais and Paice [21]. This was done by monitoring spectrophotometrically the change in absorbance at 420 nm (A_420_) related to the rate of oxidation of 1 mM 2,2′-azino-di-[3-ethylbenzothiazoline-6-sulphonate] (ABTS) in 50 mM sodium acetate buffer (pH 3.8). Assays were performed in 1 mL cuvettes at room temperature with 750 *μ*L ABTS and 250 *μ*L of enzyme extract. One unit of laccase activity was defined as the amount of enzyme that leads to the oxidation of 1 *μ*mol of ABTS per minute with a molar extinction for the ABTS radical cation (the reaction product) of *ɛ*_420 nm_ = 36000 M^−1^ cm^−1^.

#### 2.4.4. Determination of Peroxidase Activity

Total peroxidase activity was assayed according to the method of Hunter et al. [22]. Peroxidase activity was determined via oxidation of 0.24 mM 2,2′-azino-di-[3-ethylbenzothiazoline-6-sulphonate] (ABTS) buffered with 50 mM sodium acetate buffer pH 5.0 in the presence of 5 mM H_2_O_2_ at 414 nm for 5 min in a UV/Visible spectrophotometer (Unico). The reaction mixture (750 *μ*L) contained equal volume of ABTS, culture supernatant, and H_2_O_2_. One unit (U) of peroxidase activity was defined as the amount of enzyme oxidizing 1 *μ*mol ABTS per minute at pH 5.0 and 25°C with a molar extinction coefficient for the ABTS radical cation (the reaction product) of *ɛ*_414 nm_ = 31100 M^−1 ^cm^−1^.

#### 2.4.5. Determination of Protein Concentration

Protein concentration was determined by the method of Bradford [23] using bovine serum albumin (BSA) as standard. In the assay, 200 *μ*L of diluted dye reagent was pipetted into 10 *μ*L of sample solution. The mixture was then incubated at room temperature for 15 minutes to allow proper colour development. The absorbance was measured at 595 nm against blank. The specific activities of cellulase, xylanase, laccase, and peroxidase were expressed as U/mg protein

### 2.5. Investigation of Interaction of the Lignocellulolytic Enzymes Produced by* Sporothrix carnis* during Degradation of Corn Cob 

#### 2.5.1. Effect of Cultivation Time on Degradation of Corn Cob and Production Dynamics of Lignocellulolytic Enzymes by* Sporothrix carnis*

Degradation of corn cob with* Sporothrix carnis* was carried out in a shaking incubator at 155 rpm and 30°C in flask containing 100 mL of the CCB media. The initial pH of the CCB media was adjusted to 6.0 prior to autoclaving. The CCB media were inoculated with 3% inocula of seed cultures and incubated in a shaking incubator at 155 rpm. The biodegradation of corn cob and production of enzymes were monitored over 240 h at 48 h intervals. At the end of each fermentation period, the fermented medium was filtered and the total biomass consisting of fermented substrate and mycelia was measured using Whatman filter paper to evaluate the degradation of corn cob in the media. The experiments were done in triplicate. The filtrate was thereafter centrifuged at 4°C and 6000 rpm for 15 min. The supernatant obtained was used to assay for the activity of lignocellulolytic enzymes according to standard procedures described earlier. The degradation efficiency was calculated as

(1) $$\begin{array}{c} \%\;\;\text{Degradation}=\frac{\text{Initial Weight of biomass before fermentation}-\text{Final Weight of biomass after fermentation}}{\text{Initial Weight of biomass before fermention}}\times 100. \end{array}$$

#### 2.5.2. Effect of Inoculum Size on Degradation of Corn Cob and Production Dynamics of Lignocellulolytic Enzymes by* Sporothrix carnis*

Effect of varying inoculum size was determined by varying the percentage of inoculum introduced into sterile CCB media. The percentages of inocula used were 1%, 2%, 2.5%, 5%, 7.5%, and 10%. Corn cob based media (50 mL) were prepared and the pH was adjusted to 6.0. The media were autoclaved, cooled, and inoculated with 0.3 mL, 0.6 mL, 0.75 mL, 1.5 mL, 2.25 mL, and 3 mL, of the 3-day-old seed culture of* Sporothrix carnis*, respectively. The inoculated media were transferred to a shaking incubator and incubated at 155 rpm for 144 h which was the optimum incubation time obtained for production of lignocellulolytic enzymes during degradation of corn cob by* S. carnis*. At 144 h, the degradation efficiency was evaluated as described earlier and the fermentation broths were centrifuged and the supernatants were used to assay for the activities of lignocellulolytic enzymes. The experiments were done in triplicate.

#### 2.5.3. Effect of pH on Degradation of Corn Cob and Production Dynamics of Lignocellulolytic Enzymes by* Sporothrix carnis*

The effect of pH on corn cob degradation was determined by preparing the CCB media at varying pH of 4.0–10.0. CCB media (50 mL) were prepared and pH values of the media were adjusted to 4.0, 5.0, 6.0, 7.0, 8.0, 9.0, and 10.0, respectively. The media were autoclaved and inoculated with 2.5% of* Sporothrix carnis* seed culture. The inoculated media were incubated at 30°C in a shaking incubator (Stuart) 155 rpm for 144 h after which the fermentation broths were centrifuged. The supernatants were used to assay for the activity of lignocellulolytic enzymes. The degradation efficiency was determined as earlier described. The experiments were carried out in triplicate.

## 3. Results and Discussion

### 3.1. Effect of Cultivation Time on Degradation of Corn Cob and Production and Interaction of Lignocellulolytic Enzymes by* Sporothrix carnis*

**Figure 1:**
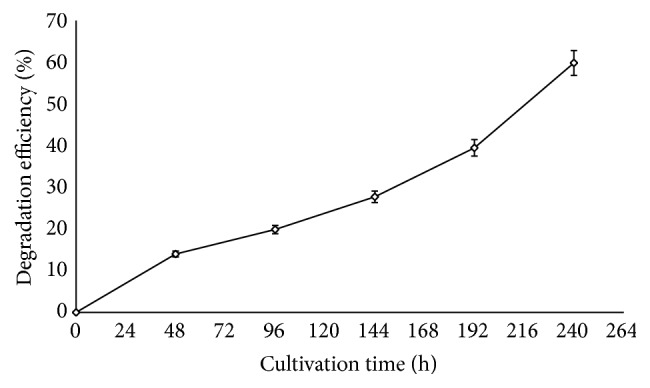
Effect of cultivation time on degradation of corn cob by* S. carnis* (error bars represent mean values and standard deviation of triplicate determination).

**Figure 2:**
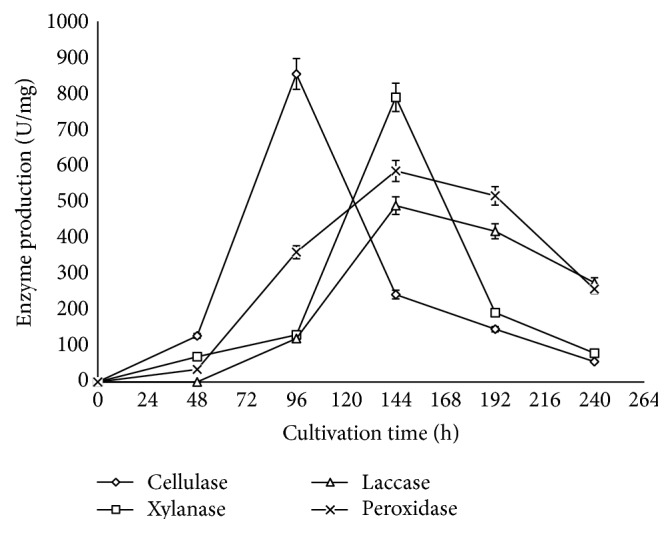
Effect of cultivation time on production of lignocellulolytic enzymes (error bars represent mean values and standard deviation of triplicate determination).

Results revealed that 14% of corn cob was degraded by 48 h of fermentation (Figure 1). The degradation was observed with the commencement of production of lignocellulolytic enzymes. This shows that enzymes catalyse the breakdown of lignocellulosic components of corn cob into metabolites that* Sporothrix carnis* utilized for growth [24]. It was observed that, as the cultivation time increased, the degradation increased to 19.87% at 96 h, 27.71% at 144 h, and 39.47% at 192 h and about 60% degradation was obtained at 240 h. The results showed that the degradation of corn cob increased with time. Analysis of enzyme production shows that the yield of cellulase was highest among the four enzymes assayed at 48 h (Figure 2). Cellulase was the first enzyme that acted on the corn cob during the degradation as 127.6 U/mg of it was produced at 48 h, although low amount of xylanase (69.68 U/mg) and peroxidase (34.6 U/mg) was obtained at this cultivation time (Figure 2). It was however surprising to observe that laccase was not detected at 48 h. This might be an indication that, in the degradation of lignocellulose, the *β*-1,4-bonds in the cellulose component are the first to be hydrolyzed which makes the other components accessible to degradation [6]. The capacity of this fungus to produce high levels of cellulase and xylanase is of importance in supplying the growing culture with a carbon source and nutrients essential for their biosynthetic activity [10].

At 96 h of cultivation, the degradation efficiency increased to 19.89% (Figure 1). At this cultivation time, it was observed that cellulase production reached a climax with maximum production of 854.4 U/mg (Figure 2). This consequently stimulated an increase in xylanase production (specific activity of 130.5 U/mg) at 96 h which was twice the activity recorded at 48 h of cultivation (Figure 2). It was observed that, as the hydrolysis of the cellulose and hemicellulose component of the corn cob increased, the lignin component became more accessible which was evident with the concerted increase in peroxidase activity. The result showed that laccase production was observed at 96 h of cultivation with specific activity of 98.2 U/mg (Figure 2). This suggests that laccase from this organism is not a primary metabolite that the fungus requires for growth as it is produced abundantly in the later phase of the degradation process [25, 26].

After 96 h, cellulase activity declined rapidly and the production of the other enzymes (xylanase, peroxidase, and laccase) increased substantially. Optimum production of these three enzymes was obtained at 144 h (Figure 2) with lignocellulose degradation of 27.7% (Figure 1). It was surprising to observe that maximum production of xylanase, laccase, and peroxidase was obtained as cellulase production declined. This indicates that the activities of the enzymes in the degradation process depend on the action of cellulase to initiate the process. It could be inferred that the rapid increase observed in the degradation is a result of the generation of many open ends through cellulose hydrolysis which enhanced accessibility to the recalcitrant lignin component of the corn cob [27]. As the cultivation time increased above 144 h, production of hydrolytic enzymes declined (Figure 2). This decline could be a result of exhaustion of glucose and xylose which are primary metabolites obtained from the degradation of corn cob by* Sporothrix carnis *required for growth. It was observed that, during this period, the yield of oxidative enzymes was still high (516.27 U/mg peroxidase; 417.8 U/mg laccase) which accounts for increase in degradation efficiency up to 39.5% and 59.81% at 192 h and 240 h of degradation, respectively.

The results obtained on the production of lignocellulolytic enzymes by* S. carnis *during corn cob degradation support the finding of Kachlishvili et al. in which it was reported that production of these enzymes was optimal at 144 h of fermentation by some basidiomycetes during fermentation of food wastes [10]. Similarly, Sharma et al. reported maximum xylanase production from* Aspergillus *sp. at 144 h of cultivation [28]. However, some fungi have been reported to produce lignocellulolytic enzymes within 120 h of cultivation, whereas some extend beyond 144 h [29–31].

### 3.2. Effect of Inoculum Size on Degradation of Corn Cob and Production and Interaction of Lignocellulolytic Enzymes by* Sporothrix carnis*

**Figure 3:**
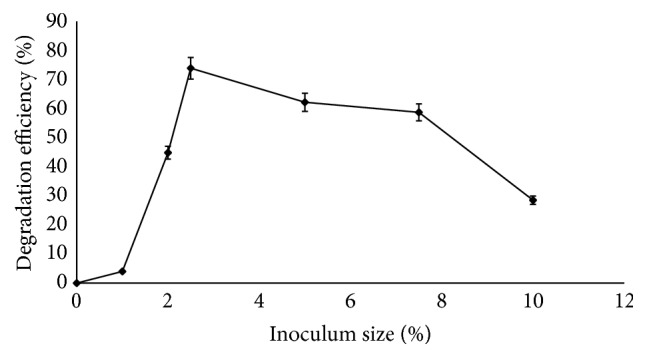
Effect of inoculum size on degradation of corn cob by* S. carnis* (error bars represent mean values and standard deviation of triplicate determination).

**Figure 4:**
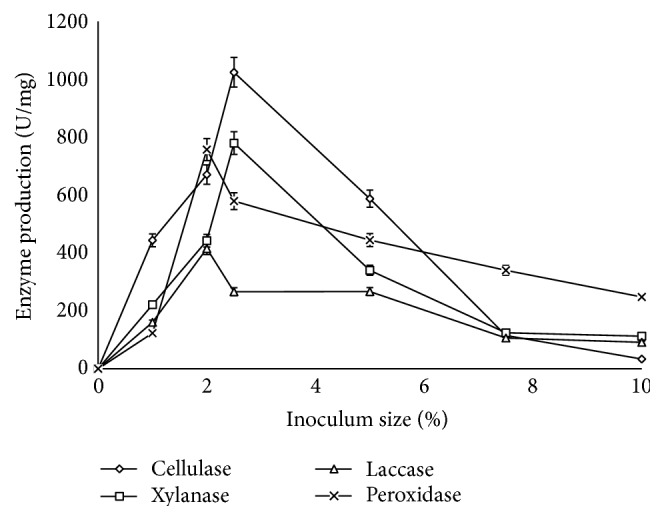
Effect of inoculum size on production of lignocellulolytic enzymes (error bars represent mean values and standard deviation of triplicate determination).

Investigation of the effect of inoculum size on corn cob degradation by* Sporothrix carnis* revealed that the microbial load had an impact on the degradation process. It was observed that the degradation efficiency of* Sporothrix carnis* increased to 73% at 144 h when 2.5% of the inoculum was used while 59.8% degradation efficiency was achieved at 240 h of fermentation when 3% of the inoculum was used (Figure 3). With the use of 1% inoculum, 4.03% degradation of the corn cob was achieved at 144 h. Degradation efficiencies of 44.83%, 62.2%, and 58.7% were obtained with 2%, 5%, and 7.5% inocula, respectively (Figure 3). The results revealed that optimum inoculum size is of key importance in degradation studies as lower inoculum size allows microbial culture to multiply at a slower rate and hence insufficient utilization of the substrate to produce metabolites. On the other hand, larger inoculum size results in rapid multiplication of cells that gets the nutrient exhausted quickly with time [24]. Surprisingly, the hydrolytic enzymes were produced optimally when 2.5% inoculum size was used with cellulase activity of 1024.22 U/mg and xylanase activity of 779.82 U/mg while 2% inoculum size supported the production of the oxidative enzymes with specific activities of 415.45 U/mg and 757.41 U/mg for laccase and peroxidase, respectively (Figure 4). Presence of abundant spores in inoculum has been reported to facilitate rapid proliferation and biomass synthesis for enzyme production as was evident with the production of cellulase and xylanase obtained in this study [32, 33].

At inoculum sizes above 2.5%, it was observed that there was a decline in the production of the lignocellulolytic enzymes which eventually resulted in rapid decline in the degradation efficiency of* S. carnis*. Previous studies have shown that, beyond a certain inoculum load, enzyme production may decrease due to the depletion of nutrients and developed oxygen tension in the medium resulting from enhanced biomass. These factors may individually or collectively result in decrease of metabolic activity [34, 35]. Various inoculum sizes have been reported to support the optimum production of these lignocellulolytic enzymes. Reports have shown that lower inoculum sizes favour the production of these enzymes as it was reported that the nutrient and oxygen levels in the fermentation media are sufficient for the growth of fungi and therefore enhanced production of lignocellulolytic enzymes [36]. Gunny et al. [33] reported maximum cellulase production from* Aspergillus terreus* with 4% inoculum. Omojasola et al. in their study reported a decline in glucose production by some fungi when cultured on pineapple waste at inoculum sizes above 6% and 8% using* A. niger* and above 4% and 6% for fermentations using* T. longibrachiatum* as a result of low yield of cellulase [37]. Niladevi et al. observed an increase in laccase production at inoculum size within 1.0–4.0% and a decrease at 5% inoculum size [38]. The increase in the yield of hydrolytic enzymes when 2.5% inoculum was used over the oxidative enzymes by* S. carnis* could be a reason for the optimum degradation obtained at this inoculum size indicating that the hydrolytic enzymes play a more prominent role in the degradation process than the oxidative enzymes.

### 3.3. Effect of pH on Degradation of Corn Cob and Production and Interaction of Lignocellulolytic Enzymes by* Sporothrix carnis*

**Figure 5:**
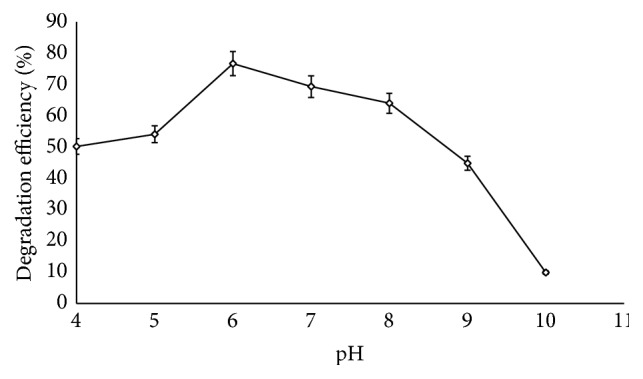
Effect of pH on degradation efficiency of corn cob by* S. carnis* (error bars represent mean values and standard deviation of triplicate determination).

**Figure 6:**
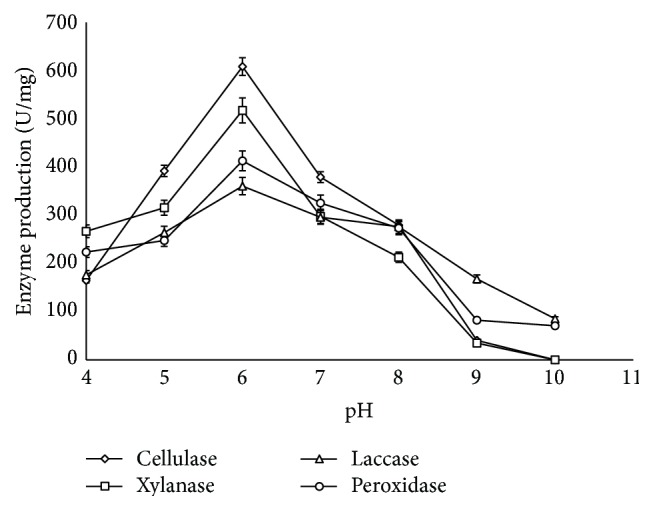
Effect of pH on production of lignocellulolytic enzymes (error bars represent mean values and standard deviation of triplicate determination).

In optimizing various conditions that influence enzymatic degradation of lignocellulosic biomass, pH was found to be a critical factor that affects the process. The result obtained showed that the degradation of corn cob by* S. carnis* increased with pH and an optimum degradation of 76.65% was obtained at pH 6.0 (Figure 5). As the process tends towards the alkaline region, the result revealed that degradation of corn cob declined. It was observed that all the lignocellulolytic enzymes were produced optimally at pH 6.0 with cellulase being the most abundant one as it had a specific activity of 608.52 U/mg closely followed by xylanase (517.6 U/mg) and peroxidase (412.89 U/mg) and the least was laccase with a maximum specific activity of 360.6 U/mg (Figure 6). As the pH increased in the alkaline region, it was observed that the production level of the enzymes decreased which resulted in lower degradation efficiency. At pH 10.0 when none of the hydrolytic enzymes was detected, the degradation efficiency was only 10%.

The overproduction of the hydrolytic enzymes under this condition as compared with the oxidative enzymes confirmed that the hydrolytic enzymes perform a primary and critical role in lignocellulose degradation [26]. Also, an optimum production of the hydrolytic and oxidative enzymes at pH 6.0 suggests the existence of synergistic interaction between the hydrolytic and oxidative enzymes which enhanced the degradation efficiency by* S. carnis*. The result revealed that degradation of lignocellulose depends more on the action of the hydrolytic enzymes to initiate the degradation enabling access to the recalcitrant lignin component during the degradation process. Furthermore, the result suggests that the mechanism of action of the oxidative enzymes depends more on the action of the hydrolytic enzymes [25, 26].

Previous reports on other ligninolytic fungi show that maximum cellulase production was in the acidic region. Maximum cellulase activity of 0.925 U/mL at a pH of 4 was observed by Acharya et al. when they used* Aspergillus niger *for the solid state fermentation of sawdust [39]. Sohail et al. reported maximum cellulase activity at a pH of 4 for* Aspergillus niger *[40]. Sugarcane waste was fermented for cellulase production via solid state fermentation using* Aspergillus niger *and* Trichoderma viride* at optimum pH of 4.5 [41]. Ilyas et al. reported optimum pH of 4.5 in their studies for cellulase production optimization [42]. Toor and Ilyas also found that cellulase was produced in higher yield in acidic region with optimum production at pH 4.0 which corresponded to the maximum degradation of cellulosic chickpea agro waste substrate [43]. Similarly, optimum pH of 4.0 and 5.0 had been reported for maximum yield of crude lignin modifying enzymes by* Coriolus hirsutus and Trametes villosa,* respectively [44]. Wen et al. reported the optimum pH of 5.0 for manganese peroxidase production from* P. chrysosporium *[45]. Similarly, Yasmeen et al. reported that acidic conditions support lignin modifying enzymes from* Schizophyllum commune* and* Ganoderma lucidum *[46]. Results obtained from this study showed that acidic condition favours the degradation process and production of lignocellulolytic enzymes by* Sporothrix carnis* is in a synergistic manner under this condition which enhanced better degradation of the corn cob under study.

## 4. Conclusion

The results from this study have given some insights into the synergistic interactions between hydrolytic and oxidative enzymes involved in the degradation of lignocellulosic biomass. The absence of any of these lignocellulolytic enzymes could adversely affect the degradation process. Improved degradation of the lignocellulose in the study was achieved by optimizing process parameters for efficient production of lignocellulolytic enzymes.
